# Health literacy and its association with antibiotic use and knowledge of antibiotic among Egyptian population: cross sectional study

**DOI:** 10.1186/s12889-024-19668-3

**Published:** 2024-09-16

**Authors:** Sally Waheed Elkhadry, Marwa Ali Haseeb Tahoon

**Affiliations:** Department of Epidemiology and Preventive Medicine, National Liver Institute, Menoufia University, Menoufia, Egypt

**Keywords:** Health literacy, Antibiotic resistance, Antibiotic use, Knowledge, Egypt, Surveys, Questionnaires

## Abstract

**Background:**

High prevalence of inappropriate antibiotic use in different sectors of the community indicates a possible strong influence of social and cultural context, which may be influenced by social determinants of health and thereby affecting individuals’ health-related behavior, including antibiotic use. And such health-related behavior is largely determined by individuals’ health literacy.

**Objective:**

The purpose of this research was to examine the relationship between the Egyptian population's health literacy, antibiotic use, and antibiotic knowledge.

**Method:**

In Egypt, researchers used a cross-sectional study design, between the period of January and March 2024 using validated questionnaires and recruited a convenient sample of adults from seven governorates representing different geographic areas, and socioeconomic and educational backgrounds with the help of the validated questionnaires the HLS-EU-Q16, a shortened version of the European Health Literacy Survey Questionnaire (derived from the World Health Organization's Antibiotic Resistance: Multi-Country Public Awareness Survey) and, familiarity with drugs and understanding of antibiotic resistance via online methods and face-to-face interviews.

**Results:**

The survey included a participation of 500 persons in total. The participants' age distribution indicated that 28.8% (*n* = 144) were aged 18–24, while 27.4% (*n* = 137) were within the 25–34 age group. Moreover, a total of 274 participants, accounting for 54.8% of the sample, described themselves as female. Significantly, 60.7% of the patients indicated acquiring antibiotics without a prescription. The evaluation of health literacy (HL) levels among the participants revealed that 36.2% had problematic HL, while 8.4% showed inadequate HL. A strong positive link was found between the scores of antibiotic knowledge and the scores of HL (*R* = 0.876; *P*-value = 0.001).

**Conclusion:**

Overall, it is imperative to implement awareness-raising curricula and public health education initiatives without delay. Furthermore, it is highly advised to implement public health awareness initiatives about the appropriate use of antibiotics, alongside national policies aimed at regulating the availability and prescription of antibiotics.

## Introduction

Health literacy is a critical determinant of individual and community health outcomes, influencing how people access, understand, and utilize healthcare information and services. It encompasses the ability to obtain, process, and apply health information to make informed decisions about health-related issues [1].

The role of health literacy (HL) in promoting health and managing sickness has been highlighted in recent years in the literature [2, 3]. The health care system is more likely to be misused by people with low health literacy [4]. The ability to read, comprehend, and critically analyze health information in order to make informed decisions about one's health is known as health literacy [5]. Health literacy impacts the way patients and doctors interact, as well as their ability to take care of themselves and navigate the healthcare system [6]. Furthermore, studies have demonstrated that information may not be enough to influence people to alter their health-related habits.

Low health literacy poses significant challenges. It can lead to misunderstandings about medical conditions and treatments, poor adherence to medications, increased hospitalizations, and higher healthcare costs. Moreover, it exacerbates health disparities, affecting vulnerable populations disproportionately [7].

In the context of antibiotic use, health literacy plays a pivotal role in shaping individuals' knowledge and behaviors regarding the appropriate use of antibiotics, which is essential for combating antibiotic resistance a global public health threat [8].

Antibiotics are powerful medications used to treat bacterial infections, but their misuse and overuse contribute significantly to the emergence of antibiotic-resistant bacteria. Misconceptions about antibiotics, such as their effectiveness against viral infections or the belief that incomplete courses are sufficient, are prevalent among populations with low health literacy levels. Such misunderstandings can lead to inappropriate antibiotic requests from healthcare providers, non-adherence to prescribed regimens, and self-medication practices, all of which exacerbate antibiotic resistance [9].

Furthermore, studies have demonstrated that information may not be enough to influence people to alter their health-related habits. Nevertheless, there is a lack of study on antibiotic use, even if skills like health literacy could be vital for people to effectively manage their own health and condition [10]. A recent American study found that parents with lower levels of education were less likely to believe in the benefits of antibiotics and were also less likely to treat their children's symptoms of what could be a viral disease with antibiotics. Concerning parents' health literacy, this matter is obviously relevant [11]. Studies looking at prescription rates frequently overlook the fact that people regularly buy antibiotics from pharmacists on their own when evaluating community antibiotic use. Additional research into the relationship between health literacy and this means of drug acquisition is warranted because of the potential links to socioeconomic status, minority ethnicity, and level of education.

Understanding the concept of antimicrobial resistance, we are unaware of any published statistics regarding the associations between the Egyptian population and the incorrect use of antibiotics and the development of antimicrobial resistance, which is a significant issue that needs to be addressed. A high rate of improper antibiotic usage across population subsets suggests that social and cultural factors, in turn impacted by socioeconomic determinants of health, play a significant role in shaping people's health-related behaviors, such as antibiotic use. And people's health literacy levels are major determinants of this kind of health-related behavior. A data gap exists on public health literacy in Egypt and how it relates to antibiotic usage, knowledge, and resistance to these drugs. The purpose of this study is to determine the relationship between the Egyptian population's health literacy and their antibiotic use, antibiotic knowledge, and antibiotic resistance awareness.

## Methods

### Design and setting

Using validated questionnaires, researchers in Egypt performed a cross-sectional study from January to March of 2024.

### Population, sample size, and sampling technique

Researchers used a convenience sample to pick people from four governorates (Menoufia, Cairo, Minya and Alexandria) in Egypt, including those in the urban, lower Egypt, Upper Egypt, and frontier governorates. Multistage random sampling was used to select four governorates (Menoufia, Cairo, Minya and Alexandria), then two districts from each governorate. Finally convenient samples were collected from each district.

Recruitment was instructed in urban and rural areas to capture a wide range of socio-economic backgrounds. The study intended to recruit participants from different educational and socioeconomic levels and not to miss rural and lower educational and socioeconomic backgrounds. "Primary education" refers to the completion of primary school, while "high education" refers to the completion of university. The individuals were recruited through face-to-face interviews conducted by trained interviewers from National Liver Institute, Menoufia University in Menoufia governorate. In other governorates, online methods such as Google Forms were used and distributed via email, WhatsApp, and other social media.

We combined multistage random sampling with convenience sampling and face to face interviewing with online methods to ensure diversity, decrease bias and to balance practicality with better represent the broader population.

Inclusion and Exclusion Criteria:

- Inclusion criteria: Egyptians over the age of eighteen were eligible for the study, while exclusion criteria were those Egyptians under eighteen, those who refused participation, those who couldn’t give informed consent, and non-Egyptians were excluded from the study.

The study was conducted among general population in Egypt. A sample size of 265 participants were predicated upon a 95% confidence interval and a 5% margin of error, taking into account the antecedently documented level of 22% pertaining to public cognizance of the concept of 'antibiotic resistance' within the confines of Egypt [12] and Egypt total estimated population as of April 2025 of 106,270,988 [13]. Lastly, the study involved a total of 500 individuals. As, we nearly duplicated the sample size half of them face to face interview and the other half self-administered online questionnaire to reach a wider range of participants.

#### Data collection questionnaire

The questionnaire was adapted from previous literature. There were three parts to our questionnaire:

- Details on the population's demographics (such as its age, gender, place of residence, marital status, and level of education);
  - *Health literacy* (with the help of the HLS-EU-Q16, a modified version of the European Health Literacy Survey Questionnaire) [14]. When it comes to health care decision-making, illness prevention, and health promotion, the 16 items that make up the HLS-EU-Q16 assess how well people are able to access, comprehend, evaluate, and apply information.
  - The HLS-EU-Q16 Questionnaire is composed of 16 questions covering three domains: healthcare (Q 1 to Q7), disease prevention (Q 8 to Q12) and health promotion (Q 13 to Q16). The Arabic version of the questionnaire was available and assessed previously among Arabic speaking migrants [15, 16].
  - The data were collected and coded as follows: ‘very easy’ and ‘fairly easy’ responses were coded as ‘1’, where ‘fairly difficult’ and ‘very difficult’ were coded as ‘0’. For each participant, a total score was calculated by summing all responses from each 16 questions ranging from 0 to 16 maximum total score. Participants were then grouped into three levels of HL: participants' scores 13 or more were considered ‘sufficient’, 9–12 ‘problematic’ and 8 ‘inadequate’ HL [15].

As well as *antibiotics usage*, understanding of antibiotics and resistance to antibiotics (derived from the WHO's Antibiotic Resistance: Multi-Country Public Awareness Survey) [12].The term “antibiotic knowledge” encompasses the following aspects: Understanding of appropriate antibiotic use, Knowledge of antibiotic resistance, Awareness of side effects, and General awareness The questionnaire is composed of 16 questions: Q1 to Q3 asking about Last antibiotic intake, how providing antibiotics, and getting advice from doctor or pharmacist about how to take the antibiotic (not included in knowledge score). The remaining 13 questions were about knowledge of antibiotics usage. The data were collected and coded as follows: each correct answer was coded as ‘1’ and otherwise (ie, not correct or ‘don’t know’ answers) a score of ‘0’. Also, 5-point Likert scale responses were summed in to two categories: if the correct answers were ‘agree’ or ‘strongly agree’, these were coded as ‘1’and if not a score of ‘0’ and contrariwise. Percentage scores were calculated for each participant. For comparative analysis, percentage scores < 50% were considered ‘poor’ and ≥ 50% were considered ‘good’ level of knowledge of antibiotics [8].

### Ethical considerations

Under protocol number (IRB number 00532/2024), the NLI ethics committee authorized this study (NLI IRB 00014014). In order to participate, every participant had to sign an informed consent form. All of them were informed of the study objectives. We informed them that we considered their answers and data confidential, Participants were under no pressure to continue participating in the study if they decided to stop at any point.

### Statistical data analysis

The responses obtained from the questionnaire were encoded and analyzed using the Statistical Package for Social Sciences (SPSS), version 22.0 (Armonk, NY: IBM Corp.). Categorical data were summarized using frequencies and proportions, while continuous variables conforming to a normal distribution were described by means and standard deviations. However, for continuous variables that did not follow a normal distribution, medians and interquartile ranges were utilized as descriptive statistics. The chi-square test was employed to evaluate the association between qualitative variables. In cases where more than 25% of the cells had an expected count lower than five, Fisher's exact test was utilized for qualitative variables. Pearson's correlation coefficient was calculated to examine the correlation (linear association) between health literacy levels and knowledge of antibiotics, given the normal distribution of the data. Statistical significance was defined as a *p*-value less than 0.05 [17, 18].

## Results

### The socio-demographic data

According to socio-demographic data, 144 (28.8%) participants their age between 18–24 years, 137 (27.4%) of them from 25–34 years, 129 (25.8%) from 35–44 years, 47 (9.4%) from 45–54 and 11 (2.2%) above 65 years. 54.8% (*n* = 274) of the participants were female, 72.8% were married (*n* = 364), 255 (51%) participants were from urban areas and 245 (49%) from rural area (About 127 people from Cairo Governorate, 98 from Alexandria Governorate, 118 from Minya Governorate, and 157 from Menoufia Governorate), and 42% (*n* = 210) highly educated (Table 1).

**Table 1:** Socio-demographics characteristics of the population participating in the research (no=500)

**Characteristics (no=500)**		**No (%)**
**Age groups **	18-24ys	144 (28.8%)
	25-34ys	137 (27.4%)
	35-44ys	129 (25.8%)
	45-54ys	47 (9.4%)
	55-64ys	32 (6.4%)
	+65ys	11 (2.2%)
**Gender **	Male	226 (45.2%)
	Female	274 (54.8%)
**Marital status **	Single	136 (27.2%)
	Married	364 (72.8%)
**Residence **	Urban	255 (51%)
	Rural	245 (49.0%)
**Education **	Illiterate	82 (16.4%)
	Primary education	208 (41.6%)
	Secondary and high education	210 (42.0%)

All data were presented as number & percentage (no, %)

### Use of antibiotics

Approximately 73.2% of participants reported having used antibiotics in the last 12 months, 22.8% have used antibiotics for more than one year and 4% had never taken antibiotics. About 60.7% of the participants did not get their antibiotics based on a prescription (about 43.3% from pharmacy without prescription and 17.4% from friends) and 21.8% did not get professional medical advice on how to take them (Table 2).

**Table 2:** Behaviors related to antibiotic use & ways of providing (*n*=500)

**Antibiotic use & ways of providing **		**No (%)**
**Last antibiotic intake **	< 1year	366 (73.2%)
	≥ 1 year	114 (22.8%)
	Never	20 (4.0)
**How do you provide antibiotics? (no=480)**	from the pharmacy after being prescribed by a doctor	185 (38.5%)
	pharmacy without prescription	208 (43.3%)
	From friend	87 (17.4%)
**Did you get advice from your doctor or pharmacist about how to take the antibiotic? (no=480)**	Yes	371 (74.2%)
	No	109 (21.8%)

### Health literacy

**Fig. 1:**
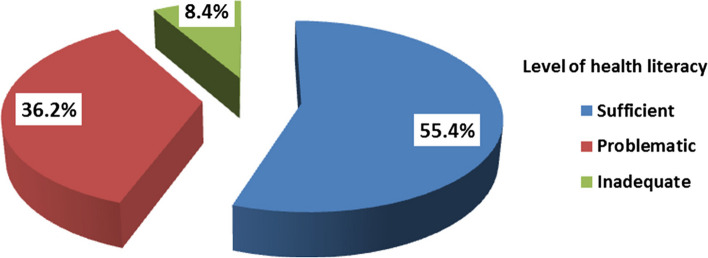
Level of health literacy (HL), *n* = 500

Around 55.4% of the subjects had appropriate HL, while 36.2% had problematic HL and 8.4% had inadequate HL (Fig. 1). The analysis did not uncover any statistically meaningful variations in health literacy proficiency across different age groups, residential locations, or marital statuses. Nonetheless, a statistically significant disparity emerged between health literacy level and gender, with 60.6% of participants demonstrating sufficient health literacy being female (*p*-value = 0.008). Additionally, a statistically significant difference was observed between health literacy levels and educational attainment, as 47.3% of participants exhibiting sufficient health literacy had achieved higher education (*p*-value = 0.019) (Table 3). The analysis did not unveil any statistically meaningful discrepancies in the duration of the participants' most recent antibiotic intake across the three levels of health literacy (*p*-value = 0.385). Conversely, 65.7% of the participants with sufficient health literacy obtained antibiotics based on a prescription, exhibiting a statistically significant difference (*p*-value = 0.001). Furthermore, 83.7% of participants with sufficient health literacy sought professional medical guidance on how to administer the antibiotics, a finding that also demonstrated statistical significance (*p*-value = 0.001) (Table 4).

**Table 3:** Background characteristics and level of health literacy among the population participating in the research

Socio-demographic characteristics	Level of health literacy, *n* = 500			*X*^2^	*P* value
	Inadequate (no = 42)	Problematic (*n* = 181)	Sufficient (no = 277)		
Age				3.5	0.474
• 18–24 years	15 (35.7%)	51 (28.2%)	78 (28.2%)		
• 35–54 years	26 (61.9%)	111 (61.3%)	176 (63.5%)		
• ≥ 55 years	1 (2.4%)	19 (10.5%)	23 (8.35)		
Gender				9.67	0.008*
• Male	19 (45.2%)	98 (54.1%)	109 (39.4%)		
• Female	23 (54.8%)	83 (45.9%)	168 (60.6%)		
Education				11.78	0.019*
• Illiterate	8 (19.0%)	33 (18.25)	41 (14.8%)		
• Primary education	25 (59.5%)	78 (43.1%)	105 (37.9%)		
• Secondary and high education	9 (21.4%)	70 (38.7%)	131 (47.3%)		
Residence				0.408	0.64
• Urban	22 (52.3%)	88 (48.6%)	145 (52.3%)		
• Rural	20 (47.6%)	93 (51.4%)	132 (47.7%)		
Marital status				0.415	0.813
• Single	13 (31.0%)	50 (27.6%)	73 (26.4%)		
• Married	29 (69.0%)	131 (72.4%)	204 (73.6%)		

Statistically significant *P*-values ≤ 0.05 are in bold with *. Chi square test (× 2) was used to test the significance. Data were presented as number & percentage (no,%)

**Table 4:** Antibiotic use and level of health literacy among the population participating in the research

Antibiotic use questions	Level of health literacy, *n* = 500			*X*^*2*^	*P* value
	Inadequate (no = 42)	Problematic (*n* = 181)	Sufficient (no = 277)		
Last antibiotic intake				4.126	0.385
• < 1 year	11 (26.2%)	46 (25.4%)	57 (20.6%)		
• ≥ 1 year	31 (73.8%)	129 (71.3%)	206 (74.4%)		
• Never	0 (0.0%)	6 (3.3%)	14 (5.1)		
How do you provide antibiotics?				8.965	**0.001**
• from the pharmacy after being prescribed by a doctor	10 (23.8%)	6 (3.3%)	169 (65.7%)		
• pharmacy without prescription	22 (52.3%)	136 (75.1%)	50 (19.4%)		
• from friend	10 (23.8%)	39 (22.3%)	38 (13.8%)		
Did you get advice from your doctor or pharmacist about how to take the antibiotic?				13.654	**0.001**
• Yes	28 (66.7%)	123 (70.3%)	220 (83.7%)		
• No	14 (33.3%)	52 (29.7%)	43 (16.3%)		

Statistically significant *P*-values ≤ 0.05 are in bold. Chi square test (× 2) was used to test the significance. Data were presented as number & percentage (no, %)

### Knowledge of antibiotics

**Fig. 2:**
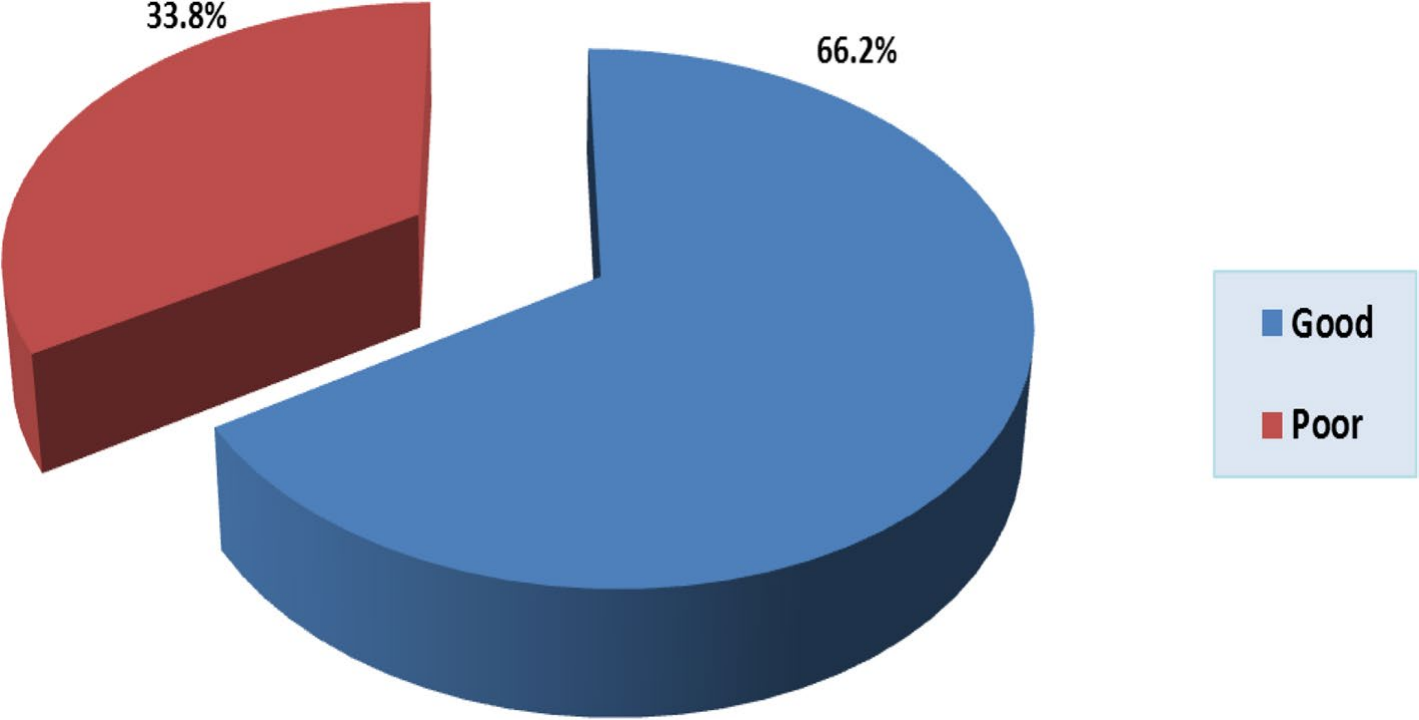
Level of knowledge of antibiotics among the study participants

A considerable proportion, approximately two-thirds (66.2%), of the participants demonstrated a commendable level of knowledge concerning antibiotics (Fig. 2). The analysis did not uncover any statistically meaningful variations in the level of antibiotic knowledge across demographic characteristics and antibiotic usage patterns, with the exception of gender. About 70.1% of the Female participants exhibited a relatively higher proportion of sound antibiotic knowledge in comparison to their male counterparts (61.5%), a discrepancy that proved statistically significant (*p*-value = 0.044) (Table 5).

**Table 5:** Levels of knowledge of antibiotics among the population participating in the research by background characteristics and antibiotic use

Characteristics	Level of knowledge of antibiotic, *n* = 500		Chi-square test	*P* value
	Good (no = 331)	Poor (*n* = 169)		
Age			4.435	0.109
• 18–24 years (no = 144)	89 (61.8%)	55 (38.2%)		
• 35–54 years (no = 313)	208 (66.5%)	105 (33.5%)		
• ≥ 55 years (no = 43)	34 (79.1%)	9 (20.9%)		
Gender			4.064	**0.044**
• Male (no = 226)	139 (61.5%)	87 (38.5%)		
• Female (no = 274)	192 (70.1%)	82 (29.9%)		
Education			4.767	0.092
• Illiterate (no = 82)	60 (73.2%)	22 (26.8)		
• Primary education (no = 208)	127 (61.1%)	81 (38.9%		
• Secondary and high education (no = 210)	144 (68.6%)	66 (31.4%)		
Marital status			0.048	0.826
• Single (no = 136)	89 (65.4%)	47 (34.6%)		
• Married (no = 364)	242 (66.5%)	122 (33.5%)		
Last antibiotic intake			6.12	0.587
• < 1 year (no = 366)	232 (63.4%)	134 (36.6%)		
• ≥ 1 year (no = 114)	82 (71.9%)	32 (28.1%)		
• Never (no = 20)	17 (85.0%)	3 (15.0%)		
How do you provide antibiotics?			0.526	0.534
• from the pharmacy after being prescribed by a doctor (no = 393)	260 (66.2%)	133 (33.8%)		
• without prescription or from friend (no = 87)	54 (62.1%)	33 (37.9%)		
Did you get advice from your doctor or pharmacist about how to take the antibiotic?			0.089	0.819
• Yes (no = 371)	244 (65.8%)	127 (34.2%)		
• No (no = 109)	70 (64.2%)	39 (35.8%)		

Statistically significant *P*-values ≤ 0.05 are in bold., Chi square test (x^2^) was used to test the significance

Data were presented as number & percentage (no, %), Percentages were calculated by row

### The correlation between health literacy proficiency and antibiotic knowledge levels

**Fig.3:**
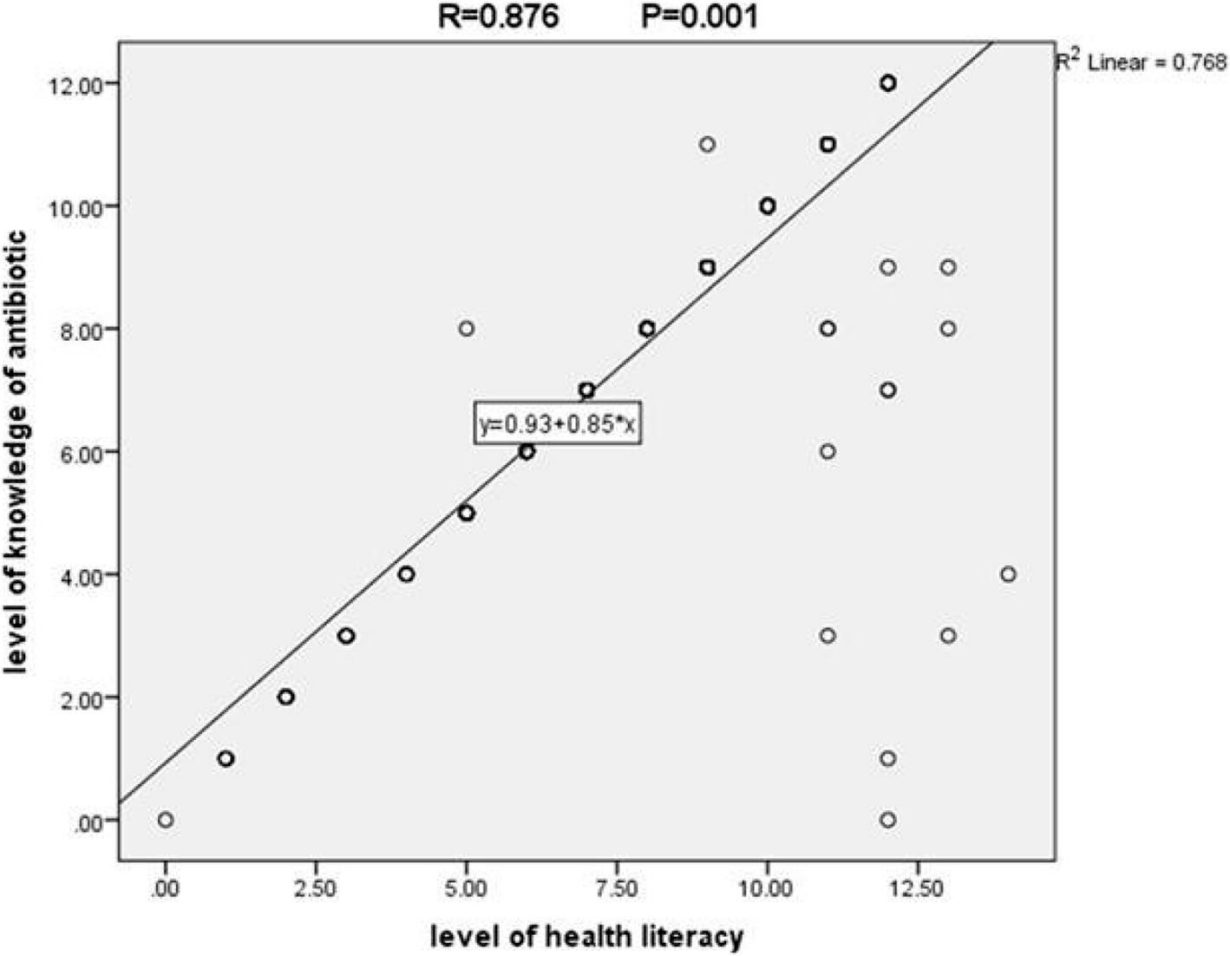
A significant positive correlation between level of health literacy and knowledge of antibiotic (*R* = 0.876, *p* = 0.001)

A significant positive correlation was observed between antibiotic knowledge scores and health literacy scores (*R* = 0.876; *P*-value = 0.001) (Fig. 3). Furthermore, a statistically significant disparity emerged between the three levels of health literacy and the level of antibiotic knowledge, with 62.2% of participants exhibiting sufficient health literacy demonstrating a relatively higher proportion of sound antibiotic knowledge (*P*-value = 0.001) (Table 6).

**Table 6:** Knowledge of antibiotics and health literacy among the population participating in the research

Level of knowledge of antibiotic	Level of health literacy, *n* = 500			*X*^*2*^	*P* value
	Inadequate (no = 42)	Problematic (n = 181)	Sufficient (no = 277)		
• Good (no = 331)	27 (8.2%)	98 (29.6%)	206 (62.2%)	20.8	**0.001**
• Poor (no = 169)	15 (8.9%)	83 (49.15)	71 (42.0%)		

Statistically significant *P*-values ≤ 0.05 are in bold. Chi square test (× 2) was used to test the significance

Data were presented as number & percentage (no, %), Percentages were calculated by row

## Discussion

This in this work, we investigated knowledge of antibiotics and their actual use, as well as the health literacy (HL) among Egyptian general population.

The results show that nearly more than half of the study population have a sufficient HL level (55.4%), while approximately one-third (36.4%) have a problematic HL level and inadequate HL level (8.4%). Approximately 36.2% of the participants had problematic HL, a proportion similar to that reported in among non-medical university students in Egypt [8], where 49% of the students had problematic HL. Also, our finding was similar with to that reported in a study among outpatient clinics attendees at ASU Hospitals (46.7% of them had problematic HL) [19]. However, levels of sufficient HL among students in this study (54.2%) were higher than levels reported in outpatient clinic attendees (18.9%) [19], and also to those reported in a population-based study in Italy, where 33% had sufficient HL [20]. However, sufficient HL in this study were lower than those reported among university students in Lithuania, [21] where the majority of students (70%) attended health education courses and two-thirds of the students (67%) had sufficient HL. This might indicate the vital role of dedicated courses in raising HL levels.

The study found an association between HL overall and socio-demographic factors, gender, and participant's level of Education with significant association. Participant's high level of education is associated with high HL scores (47% of participants with sufficient HL level were highly educated), and these findings go with other HL evidence from the Eastern Mediterranean region [22], United States [23], and Southeast Asia [24]. There was statistical significance difference between HL level and gender as 60.6% of participants with sufficient HL were female (*P* value = 0.008) which is proved by many other studies [25, 26].

More than two-thirds (73.2%) of the participants had taken antibiotics during the previous 12 months. This percentage is higher than that of the general public in low-income countries (e.g., Egypt, Sudan, and Indonesia), as reported by a WHO multicounty survey [12].

Self-medication of antibiotics involves obtaining them without a prescription to treat self- diagnosed symptoms or conditions [27]. Self-medication of antibiotics was common (43.3%) in all participants regardless of their level of HL. A similar rate (39.9%) was reported recently among non-medical university student in Egypt [8], also university students in the UAE (38.2%) [28], SriLanka (38.6%) [29], China (33.0%) [30], and in an earlier household study in Jordan (39.5%) [31]. These findings underscore the importance of conducting additional studies into the reasons for self-medication in order to increase public awareness and guide practice among the general public and healthcare professionals.

In our study, 62% of the participants with sufficient HL had good knowledge of antibiotic. This comes in line with another study where knowledge scores were significantly higher for those with adequate health literacy than those with inadequate health literacy [32], suggesting that overall health literacy is a key focus that crosses all disciplines.

According to the findings of this study, lack of knowledge of antibiotic use was more prevalent among male participants. This could be because women are more likely to use primary healthcare services, are more experienced with antibiotic treatment, and take greater responsibility for their family’s health [33, 34].

In our study, There was a significant positive correlation between scores of knowledge of antibiotic and scores of HL (*R* = 0.876; *P* value = 0.001). Therefore, approaches sensitive to health literacy can improve the knowledge of the general population [35]. In addition to increasing awareness and knowledge of antibiotics, competencies such as health literacy should be targets of intervention, especially in the primary health care setting. When people are ill, they tend more to take risks and to accept side effects, even if they know the expected benefits are low [36].

Regarding our finding: improving health literacy regarding antibiotic use is essential for combatting antibiotic resistance. By empowering individuals with knowledge about the appropriate use of antibiotics and the risks associated with misuse, we can promote responsible antibiotic stewardship and contribute to preserving the effectiveness of antibiotics for future generations. Public health efforts in Egypt and globally must continue to prioritize education and awareness initiatives to address this critical health issue effectively.

The main limitations of this study were that the sample size of 500 could result in an underrepresentation of the population as the researchers could not collect more data on non-respondents to address the possibility of selection bias as they were either out of reach or refused to participate in the study by any means. Also, convenience sampling method is considered one of the study limitation, but we addressed some steps in our research to make a convenience sampling method less biased consider the following strategies: we combined multistage random sampling with convenience sampling and face to face interviewing with online methods to ensure diversity, decrease bias and to balance practicality with better represent the broader population. Also, the cross-sectional nature of the study cannot allow causal associations between students’ HL and use of antibiotics, knowledge of antibiotics. Therefore, Future studies using analytical designs such as longitudinal, or case–control studies could help provide robust evidence for causal associations. There was an over-representation of younger respondents that did not perfectly represent the target population according to Egyptian’s census administrative records (CAPMAS) [13]. Therefore, the findings of this study could fall short in representativeness. Moreover, participants who might be concerned with the antibiotic topic could be more likely to take the survey, which might lead to self-selection. Moreover, the impact of chronic diseases on escalating antibiotic consumption was not a focus of this study.

The study strength was that our study was the first in Egypt to measure the HL levels among the general adult population and address its possible determinants and its relation with antibiotic use. Also, the survey items were adapted from previously tested questionnaires in different populations and in Egypt: The Health Literacy Survey (HLS-EU-Q16) and the WHO Antibiotic Resistance: Multi-Country Public Awareness Survey.

## Conclusion

In conclusion, addressing low levels of health literacy and combating antibiotic misuse are critical priorities for public health officials in Egypt and globally. Effective strategies include comprehensive education campaigns, targeted healthcare provider training, strengthened regulatory measures, community engagement, and leveraging technology for outreach. By implementing these measures collaboratively and continuously evaluating their impact, we can enhance health literacy, promote responsible antibiotic use, and mitigate the growing threat of antibiotic resistance. These efforts are essential for safeguarding public health and ensuring sustainable healthcare practices for future generations.

## Data Availability

Any interested party may request access to the datasets used in this work by contacting the corresponding author.
